# Serum IL-17, IL-23, and TGF-**β** Levels in Type 1 and Type 2 Diabetic Patients and Age-Matched Healthy Controls

**DOI:** 10.1155/2014/718946

**Published:** 2014-06-04

**Authors:** Azam Roohi, Mina Tabrizi, Farzaneh Abbasi, Asal Ataie-Jafari, Behrouz Nikbin, Bagher Larijani, Mostafa Qorbani, Alipasha Meysamie, Hossein Asgarian-Omran, Bahram Nikmanesh, Arezou Bajouri, Novin Shafiey, Akram Maleki

**Affiliations:** ^1^Department of Molecular Medicine, School of Advanced Technologies in Medicine, Tehran University of Medical Sciences, Tehran 1417755469, Iran; ^2^Endocrinology and Metabolism Research Center (EMRC), Dr. Shariati Hospital, Tehran University of Medical Sciences, Tehran 1411413137, Iran; ^3^Department of Immunology, School of Public Health, Tehran University of Medical Sciences, Tehran 1417613151, Iran; ^4^Department of Medical Genetics, School of Medicine, Tehran University of Medical Sciences, Tehran 1417613151, Iran; ^5^Department of Immunology, School of Medicine, Tehran University of Medical Sciences, Tehran 1417613151, Iran; ^6^Department of Public Health, Alborz University of Medical Sciences, Karaj 3139614171, Iran; ^7^Department of Epidemiology, Iran University of Medical Sciences, Tehran 1449614535, Iran; ^8^Department of Community and Preventive Medicine, School of Medicine, Tehran University of Medical Sciences, Tehran 1417613151, Iran; ^9^Department of Immunology, School of Medicine, Mazandaran University of Medical Sciences, Sari 4817844718, Iran; ^10^Department of Pathology, Children's Medical Center Hospital, Tehran University of Medical Sciences, Tehran 1419733151, Iran; ^11^Farmanfarmaian Health Center, Tehran University of Medical Sciences, Tehran 1318618311, Iran

## Abstract

Type 1 diabetes is recognized as an autoimmune inflammatory disease and low grade inflammation is also observed in type 2 diabetic patients. Interleukin 17 (IL-17) is a new player in inflammation. Th17 cells, as the main source of IL-17, require transforming growth factor **β** (TGF-**β**) and interleukin 23 (IL-23). The aim of this study was to investigate serum IL-17, IL-23 and TGF-**β** levels in diabetic patients and controls. In this case-control study, serum levels of IL-17, IL-23, and TGF-**β** were measured in 24 type 1 diabetic patients and 30 healthy controls using the ELISA method. Simultaneously, the same methodology was used to compare serum concentration of these three cytokines in 38 type 2 diabetic patients and 40 healthy controls. There was no significant difference between serum levels of IL-17 and IL-23 cytokines between cases and controls. However, TGF-**β** was significantly lower in type 1 diabetic patients (*P* < 0.001). Serum IL-17 and IL-23 levels demonstrate no association with type 1 and type 2 diabetes, but, in line with previous studies, TGF-**β** levels were lower in type 1 diabetic patients.

## 1. Introduction

Growing medical literature refers to increasing incidence of type 1 diabetes (T1D) [1]. T1D is recognized as an autoimmune disease [2]. Due to immune mediated pancreatic *β*-cell destruction, *β*-cell mass and function are decreased [3] and insulin dependence occurs. It seems both immune cells [4] and inflammatory mediators such as cytokines have roles in *β*-cell destruction. Alteration of inflammatory mediator levels like interleukin 1 beta (IL-1*β*), interleukin 6 (IL-6), tumor necrosis factor alpha (TNF-*α*), and C-reactive protein (CRP) is a characteristic of T1D [5–7]. Evidence is also accumulating to suggest low-grade systemic inflammation in type 2 diabetes (T2D) in which concentration of inflammatory cytokines such as IL-1*β* increases. IL-6 has been inferred as the other player in developing insulin resistance and T2D [8]. It seems inflammation is a common feature of type 1 and type 2 diabetic patients.

A newly described inflammatory cytokine, interleukin 17 (IL-17A), was discovered as CTLA-8 in 1993 [9] and it took about 10 years to identify the cellular source of this cytokine to be Th17 cells [10]. Increasing evidence has indicated an established role of Th17 cells in different autoimmune diseases such as systemic psoriasis, rheumatoid arthritis, inflammatory bowel disease, lupus erythematosus, and multiple sclerosis [11]. Data obtained from human T1D studies and mouse models suggest involvement of Th17 cells and IL-17 in T1D pathogenesis. Marwaha et al. [12] have shown increased proportion of a CD4+ T-cell subset secreting higher levels of IL-17 in subjects with new-onset T1D. Application of a neutralizing anti-IL-17 in nonobese diabetic mice (NOD) by Emamaullee et al. prevented diabetes, suggesting Th17 cell involvement in T1D pathogenesis [13].

Development of a Th17 cell population depends on a combination of cytokines. One such cytokine is transforming growth factor beta (TGF-*β*). Human transforming growth factor-*β*1 (TGF-*β*1) belongs to a protein family including a large number of highly conserved and structurally related proteins. Members of this family are involved in tissue homeostasis and embryogenesis [14]. TGF-*β*1 is a multifunctional cytokine with context dependent actions. It may exert either immunosuppressive or inflammatory effects. Both arms of the immune system are inhibited by regulatory effects of this cytokine [15]. This cytokine plays a major role in tolerance maintenance through controlling survival, proliferation, and differentiation of T lymphocytes. TGF-*β*1 is also able to inhibit B-cell proliferation or block B-cell activation by inducing apoptosis [16]. TGF-*β*1 is also recognized as a proinflammatory cytokine due to its involvement in Th-17 cell differentiation. In 2006, three different groups [17–19] demonstrated Th17 development by TGF-*β* in combination with IL-6. In this context, significance of TGF-*β* is controversial. While the study conducted by Veldhoen et al. [18] indicated that the antagonistic effect of TGF-*β* on development of Th1 and Th2 was not the only role that this cytokine played in Th17 development, Das et al. [20] believe there is an indirect role for this cytokine in Th17 differentiation. The second effective cytokine in TH17 cell development is interleukin 23 (IL-23). It seems IL-23 is not involved in initiation of Th17 differentiation since it has no receptor on naive T cells. However, its upregulation after the initiation phase suggests its important role for maintenance of Th17 cells [21].

To clarify whether serum levels of IL-17 demonstrate a change in T1D and T2D, we compared its concentration in the sera of 24 type 1 and 38 type 2 diabetic patients and age-matched healthy controls. Serum concentrations of IL-23 and TGF-*β* were also measured in the same group because development and maintenance of Th17 cells, as the main source of IL-17, require these two cytokines.

## 2. Materials and Methods

### 2.1. Participants

Blood from 24 diabetic patients, aged 6 to 30 years, was collected at the Diabetes and Metabolic Diseases Clinic, and serum samples of 30 healthy subjects in the same age groups were collected at the Children Medical Center Hospital and Farmanfarmaian Health Center, Tehran University of Medical Sciences (TUMS). Children were brought to the hospital for preschool health monitoring plan or prior to circumcision. Healthy children were chosen based on lack of prior medical history, lack of prescribed medication use, and normal test results. Adults were referred to the health center for annual occupational monitoring. Healthy adults lacked prior medical history or medication use (prescription or otherwise) and had normal test results (Table 1). Subsequent to selection of our healthy control group, C-reactive protein test was conducted. T1D was diagnosed based on American Diabetes Association (ADA) criteria. T1D patients were questioned in detail about any T1D complications or any other pathology other than T1D. Patients suffering from T1D complications or any other pathology were excluded from the study. The only medication taken by type 1 diabetic patients in our study was insulin. Study subjects' background information such as age, weight, and height was recorded.

Blood from 38 T2D patients and 40 adult healthy controls, aged 30 to 65 years, was taken from Farmanfarmaian Health Center, Tehran University of Medical Sciences (TUMS). These subjects were referred to the health center for annual occupational monitoring, participated in the diabetes screening project, or required periodical biochemistry tests for monitoring purposes. Healthy controls were chosen according to the above mentioned criteria (Table 2). T2D patients were recruited based on their fasting blood glucose and medical history. All diabetic patients with diabetic complications, diabetes duration of more than five years, or any other pathology other than T2D were excluded from the study. Eight of the T2D patients used metformin and/or glibenclamide.

Serum samples were collected and stored at −70°C and biochemical tests were done for all subjects. Fasting blood glucose (FBS), total cholesterol, high-density lipoprotein cholesterol (HDL), and triglyceride (TG) were measured using AutoAnalyzer (Hitachi902, Japan). Using a commercial kit (Parsazmun, Iran), low-density lipoprotein (LDL) was measured. This study was approved by the ethics committee of TUMS and informed consent was obtained from all participants.

### 2.2. Cytokine Assays

Serum concentrations of IL-17, IL-23, and TGF-*β* were measured using commercially available enzyme-linked immunosorbent assay kits (eBioscience ELISA kits, USA). Sensitivity of the kits for these cytokines was 4 picogram per milliliter (pg/mL) for IL-17, 15 pg/mL for IL-23, and 60 pg/mL for TGF-*β*. In brief, Maxisorp immunoplates (Nunc, Denmark) were coated with monoclonal antibodies (mAb) specific for IL-17, IL-23, and TGF-*β*. Then, serum samples and standards were added and serum cytokines were detected using biotinylated mAb specific for IL-17, IL-23, and TGF-*β* followed by addition of streptavidin-horseradish peroxidase and color development. Activation of serum TGF-*β* was conducted according to the manufacturer's instructions. Absorbance was read at 450 nm. Using standard curves, the values were expressed in pg/mL.

### 2.3. Statistical Analysis

All data are presented as mean ± SD unless otherwise stated. Normal distribution of type one diabetes data was assessed by Kolmogorov-Smirnov test. To compare continuous variables between groups, Student's* t*-test and Mann-Whitney* U* test were applied. In all analyses, *P* values < 0.05 were considered as statistically significant. All statistics were done using SPSS for windows version 15.

## 3. Results

This study was conducted to investigate IL-17 levels in serum of patients with T1D and T2D. Serum concentrations of TGF-*β* and IL-23 were also defined, since development and maintenance of Th17 cells, as the main source of IL-17, require these two cytokines.

Background information and biochemical characteristics of all participants are summarized in Tables 1 and 2. In T1D, serum FBS level was the only biochemical factor showing significant difference between case and control groups (*P* < 0.001). In T2D, all biochemical factors except LDL were significantly different between case and control groups.

**Figure 1:**
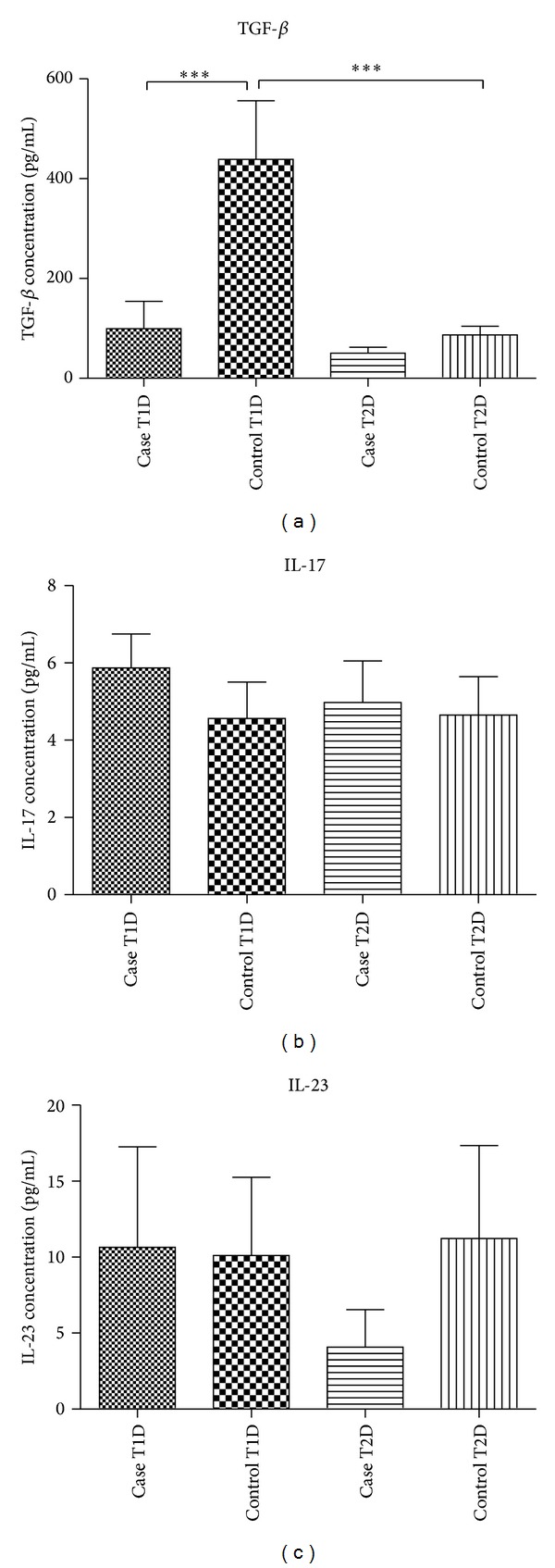
Serum TGF-*β*, IL-17, and IL-23 levels in T1D and T2D patients and healthy controls. TGF-*β* levels were significantly lower in T1D patients than controls. Comparison of TGF-*β* level between two control groups indicated it was lower in the T2D control group (***P < 0.001). Data are presented as mean ± SEM.

Serum levels of IL-17 were detectable in most samples while concentration of TGF-*β* and especially IL-23 in many sera was lower than the detection limit of the kits and in statistical analysis they have been considered to be zero since signal values for these samples were as low as signal values for blank wells during the test. Results are summarized in Figure 1 and Tables 3 and 4.

Statistical analysis showed a significant difference between serum levels of TGF-*β* in type one diabetic patients and healthy controls (*P* < 0.001). No statistical difference was observed in terms of IL-17 and IL-23 serum levels between the two groups (Tables 3 and 4). Intergroup statistical analysis was not possible due to small sample size. Comparison between healthy controls of the two groups (type one and type two diabetes) indicated a statistically significant difference between serum levels of TGF-*β* (*P* < 0.001).

## 4. Discussion

In the present study, comparison of IL-17 and IL-23 levels in serum of diabetic patients compared with healthy controls showed no significant difference while TGF-*β* level was lower in serum of type 1 diabetic patients. Decreased levels of TGF-*β* gene expression and protein secretion in T1D have been reported previously [22–26]. In the current study, TGF-*β* levels were detectable in almost 70% of healthy controls and 20% of type 1 diabetic patients. This can be a reflection of overall reduced levels of TGF-*β* in the disease state as supported by our statistical analysis. Interestingly, glucose affects TGF-*β* levels. Hyperglycemia stimulates TGF-*β* expression in different cell types such as macrophages [27], human mesenchymal stem cells [28], and mouse mesangial cells [29]. Elevated levels of TGF-*β* gene expression have been reported in patients with type 2 diabetes [26], and this is in line with effect of high concentrations of glucose on different cells. Regarding the above mentioned findings, reduced levels of TGF-*β* in T1D seem intriguing. Different cell types such as dendritic cells and naturally occurring regulatory T cells (nTregs) can produce TGF-*β* under different immunological conditions. TGF-*β* is an essential cytokine for nTreg development and function [30]. nTregs have a central role in prevention of T1D development and a major mechanism applied to delay T1D onset by nTregs is TGF-*β* secretion [31]. Functional defect related to signaling pathways leading to TGF-*β* production in these cells or other TGF-*β* sources in T1D may be the reason for the reduced levels of the cytokine. It seems that this fundamental change cannot be affected by high concentrations of glucose.

Our data indicated that serum TGF-*β* levels were higher in the T1D control group in comparison with the T2D control group. While T1D mainly strikes children and younger adults, T2D is commonly observed in adulthood. To exclude the age related variations, two age-matched healthy control groups with mean age of 18.53 and 34.98 years were separately selected for T1D and T2D patients, respectively. The mean concentration of TGF-*β* was significantly higher in T1D controls (younger controls) in comparison to other three groups including T1D patients, T2D patients, and T2D control groups (Figure 1). T1D and its control group were matched for several factors (Table 1) including age and the sole documented criteria for exception were FBS as a marker of diabetes. Due to the fact that we had tried to control measurable factors, it appeared logical to suspect that the significant difference observed in the level of serum TGF-*β* could be related to diabetes and this finding was in accordance with previous observations [22–26].

Comparing the two control groups indicated that the only significant difference between them was their age. The efficiency of the immune system declines with age. This gradual systemic failure has a multifactorial etiology including genetic and environmental factors. In the year 2000, Franceschi et al. [32] coined “Inflammaging” to refer to a proinflammatory status which is a main characteristic of aging. Several epidemiologic studies have established an association between aging and some inflammatory markers specially IL-6, TNF-*α*, and CRP [33]. A research conducted by McFarlane et al. [34] indicated that age-related inflammatory changes including rise in the level of CRP, IL-6, and ratio of IL-6 to interleukin 10 (IL-10) occur in baboons. On the other hand, Willis et al. [35], using a baboon model, have shown that serum TGF-*β* reduction is associated with age in female baboons.

These findings give rise to questions regarding the level of regulatory cytokines such as TGF-*β* in aging. It would be interesting to know if there is any change in TGF-*β* level during human lifespan. If so, can this change affect inflammaging? Lin et al. [36] found that TGF-*β* level reduction was age dependent and this reduction was inversely associated with age. Since their study group aged between 40 and 80 years, they could not follow TGF-*β* reduction trend in people aged below 40 years. Our data indicated that this trend can be observed in younger people and this finding is in line with results obtained by Okamoto et al. [37] who compared serum TGF-*β* levels between healthy subjects aged between 1 and 14 years and 21 and 67 years. Inverse association between TGF-*β* and age is controversial since this association was not observed by Grainger et al. [38]. Reduction of IL-10 as an anti-inflammatory cytokine in healthy old people has also been reported by Saurwein-Teissl et al. [39] who underscored an imbalance of pro- and anti-inflammatory cytokines in elderly people. This inconsistency in cytokine network may lead to age-related diseases with low grade inflammation such as diabetes.

Increasing data suggest a pathogenic role for Th-17 cells in autoimmune and inflammatory diseases. Role of these cells in T1D pathogenesis has been investigated by several groups. Bending et al. [40] believe Th17 cells play their role in disease through their plasticity towards a Th-1 like profile. A study by Martin-Orozco et al. [41] in NOD mice has shown that Th-17 cells play a role in inflammation of pancreas; however, for T1D induction, conversion of these cells to Th-1 cells is necessary. Other mechanisms have also been suggested to shed light on the role of these cells in T1D. It seems that increased number of Th-17 cells results in an imbalance between nTregs and effector T cells and autoimmunity [42]. We could detect IL-17 in most tested sera. Observation of no significant difference between cases and controls may be consistent with the above mentioned findings and there is lack of evidence for a role that these cells may play in T1D and T2D systemic inflammation.

In our study, IL-23 serum levels were undetectable in most cases and controls and there were no significant differences between serum concentrations in diabetic patients and healthy controls. Langrish et al. [43] refer to the role of IL-23 in Th-17 development and its indirect effect in autoimmune inflammatory diseases. This role has been investigated in a recent study by Mus et al. [44], which have shown IL-23 cytokine effect on promotion of Th-17 cell development in autoimmune experimental arthritis. Evidence for IL-23 involvement in Th-17 cell maintenance has also been presented [21]. Destructive effect of IL-23 administration on pancreatic *β*-cells leading to hyperglycemia in a mouse model [45] refers to a possibly direct role of IL-23 in autoimmune diabetes. Our goal of IL-23 serum level measurement was to detect any change in the cytokine concentration in diabetic patients' sera. Regarding the number of samples with cytokine levels below the detection limit, it seems that it is better to use a more sensitive method for the cytokine assay in serum samples. In the present study, the difference between serum levels of this cytokine in healthy subjects and T1D patients was close to being significant (*P* = 0.08). Abbasi et al. [26] have shown upregulation of IL-23 gene in unstimulated peripheral blood mononuclear cells (PBMCs) in T1D. It appears a larger sample size and a more sensitive method should be applied to study the role of this cytokine in T1D.

A minor point in our study was that serum LDL levels in T2D patients were similar to healthy control LDL levels. It is common to observe elevated levels of serum TG and decreased levels of HDL in T2D patients while LDL shows no change or even reduction due to the dysregulation of lipoprotein lipase which is involved in LDL synthesis [46].

In summary, our results suggest that serum IL-17 and IL-23 levels were not affected in diabetic patients whereas TGF-*β* was significantly decreased in T1D. Although the present study does have weaknesses which may be recognized in primary studies of novel entities, to the best of our knowledge, this is one of few studies so far conducted to elucidate the potentially significant role IL-17 and IL-23 could play in diabetes in humans. Though ELISA is a common method to measure cytokine levels, it appears that it is not sensitive enough for measurement of IL-23 in serum. Low sensitivity of the applied technique in the present study was not sufficiently rigorous to allow definitive illustration of a significant relationship or lack of a significant relationship between these two potentially important cytokines and diabetes. Further efforts should be devoted to finding more sensitive methods to assay serum cytokine levels to clearly delineate possible systemic involvement of these cytokines in diabetes. Findings in this study show that the difference between T1D patients and their age-matched controls in terms of serum TGF-*β* concentration is mostly related to the disease status because patients and controls have been matched for notable criteria such as age. Meanwhile, the higher concentration of TGF-*β* in younger control individuals compared to the older controls seems to be most logically an age-related issue (if for now only giving consideration to measurable factors) and evidence is accumulating that physiological TGF-*β* concentrations show reduction later in life. Association between TGF-*β* and age is controversial and further studies/data to clarify this possible relationship can be enlightening. Also more investigations are necessary to clarify the mechanism of TGF-*β* reduction in a hyperglycemic milieu.

## Figures and Tables

**Table 1:** Type 1 diabetes study subjects characteristics.

Variables	Case	Control
	*n* = 24	*n* = 30
Age (years)	17.96 ± 7.29	18.53 ± 8.38
Sex (M/F)	9/15	18/12
BMI (kg/m^2^)	21.09 ± 4.04	22.6 ± 3.39
FBS (mg/dL)	180.38 ± 99.19	88.3 ± 8.14
Chol (mg/dL)	160.88 ± 39.11	163.35 ± 16.31
TG (mg/dL)	87.69 ± 32.48	97.76 ± 32.46
HDL (mg/dL)	53.44 ± 16.61	50.82 ± 8.52
LDL (mg/dL)	86.5 ± 26.35	88.24 ± 14.32

M: male, F: female, BMI: body mass index, FBS: fasting blood glucose, Chol: cholesterol,

TG: triglyceride, HDL: high-density lipoprotein, and LDL: low-density lipoprotein.

Data are presented as mean ± SD.

**Table 2:** Type 2 diabetes study subjects characteristics.

Variables	Case	Control	*P * value
	*n* = 38	*n* = 40	
Age (years)	51.14 ± 8.63	34.98 ± 11.39	0.001
Sex (M/F)	19/19	22/18	
BMI (kg/m^2^)	27.39 ± 4.32	24.58 ± 4.14	0.007
FBS (mg/dL)	155.21 ± 55.6	86.12 ± 7.44	0.001
Chol (mg/dL)	190.59 ± 44.63	167.63 ± 16.92	0.01
TG (mg/dL)	234.57 ± 264.72	98.73 ± 36.93	0.002
HDL (mg/dL)	44.5 ± 13.03	52.48 ± 9.83	0.003
LDL (mg/dL)	101.42 ± 27.29	93.15 ± 17.23	0.1

M: male, F: female, BMI: body mass index, FBS: fasting blood glucose, Chol: cholesterol,

TG: triglyceride, HDL: high-density lipoprotein, and LDL: low-density lipoprotein. Data are presented as mean ± SD.

**Table 3:** Cytokine serum levels in cases and controls of type one diabetes.

Cytokine	Case	Control	*P* value
	Median (IQR)	Median (IQR)	
IL-17	4.93 (7.37)	2.61 (7.87)	0.15
IL-23	0 (4.47)	0.70 (9.1)	0.08
TGF-*β*	0 (0)	184.52 (627.22)	0.001

Interquartile range (IQR): 75th–25th.

**Table 4:** Cytokine serum levels in cases and controls of type two diabetes.

Cytokine	Case	Control	*P* value
	Mean ± SD	Mean ± SD	
IL-17	6.61 ± 4.97	6.22 ± 4.64	0.8
IL-23	15.2 ± 4.07	38.22 ± 10.94	0.3
TGF-*β*	50.46 ± 72.62	84.76 ± 72.62	0.1

## References

[1] DIAMOND Project Group (2006). Incidence and trends of childhood Type 1 diabetes worldwide 1990–1999. *Diabetic Medicine*.

[2] Alberti KG, Zimmet PZ (1998). Definition, diagnosis and classification of diabetes mellitus and its complications. Part 1: diagnosis and classification of diabetes mellitus provisional report of a WHO consultation. *Diabetic Medicine*.

[3] Rowe PA, Campbell-Thompson ML, Schatz DA, Atkinson MA (2011). The pancreas in human type 1 diabetes. *Seminars in Immunopathology*.

[4] Willcox A, Richardson SJ, Bone AJ, Foulis AK, Morgan NG (2009). Analysis of islet inflammation in human type 1 diabetes. *Clinical and Experimental Immunology*.

[5] Lechleitner M, Koch T, Herold M, Dzien A, Hoppichler F (2000). Tumour necrosis factor-alpha plasma level in patients with type 1 diabetes mellitus and its association with glycaemic control and cardiovascular risk factors. *Journal of Internal Medicine*.

[6] Chase HP, Cooper S, Osberg I (2004). Elevated C-reactive protein levels in the development of type 1 diabetes. *Diabetes*.

[7] Dogan Y, Akarsu S, Ustundag B, Yilmaz E, Gurgoze MK (2006). Serum IL-1*β*, IL-2, and IL-6 in insulin-dependent diabetic children. *Mediators of Inflammation*.

[8] Fève B, Bastard JP (2009). The role of interleukins in insulin resistance and type 2 diabetes mellitus. *Nature Reviews Endocrinology*.

[9] Rouvier E, Luciani M-F, Mattei M-G, Denizot F, Golstein P (1993). CTLA-8, cloned from an activated T cell, bearing AU-rich messenger RNA instability sequences, and homologous to a herpesvirus Saimiri gene. *Journal of Immunology*.

[10] Harrington LE, Hatton RD, Mangan PR (2005). Interleukin 17-producing CD4^+^ effector T cells develop via a lineage distinct from the T helper type 1 and 2 lineages. *Nature Immunology*.

[11] Maddur MS, Miossec P, Kaveri SV, Bayry J (2012). Th17 cells: biology, pathogenesis of autoimmune and inflammatory diseases, and therapeutic strategies. *The American Journal of Pathology*.

[12] Marwaha AK, Crome SQ, Panagiotopoulos C (2010). Cutting edge: increased IL-17-secreting T cells in children with new-onset type 1 diabetes. *Journal of Immunology*.

[13] Emamaullee JA, Davis J, Merani S (2009). Inhibition of Th17 cells regulates autoimmune diabetes in NOD mice. *Diabetes*.

[14] Derynck R, Miyazono K, Derynck R, Miyazono K (2008). TGF-beta and the TGF-beta family. *The TGF-Beta Family*.

[15] Prud’homme GJ (2007). Pathobiology of transforming growth factor *β* in cancer, fibrosis and immunologic disease, and therapeutic considerations. *Laboratory Investigation*.

[16] Li MO, Wan YY, Sanjabi S, Robertson A-KL, Flavell RA (2006). Transforming growth factor-*β* regulation of immune responses. *Annual Review of Immunology*.

[17] Bettelli E, Carrier Y, Gao W (2006). Reciprocal developmental pathways for the generation of pathogenic effector TH17 and regulatory T cells. *Nature*.

[18] Veldhoen M, Hocking RJ, Atkins CJ, Locksley RM, Stockinger B (2006). TGF*β* in the context of an inflammatory cytokine milieu supports de novo differentiation of IL-17-producing T cells. *Immunity*.

[19] Mangan PR, Harrington LE, O’Quinn DB (2006). Transforming growth factor-*β* induces development of the T H17 lineage. *Nature*.

[20] Das J, Ren G, Zhang L (2009). Transforming growth factor beta is dispensable for the molecular orchestration of Th17 cell differentiation. *The Journal of Experimental Medicine*.

[21] Boniface K, Blom B, Liu Y-J, de Waal Malefyt R (2008). From interleukin-23 to T-helper 17 cells: human T-helper cell differentiation revisited. *Immunological Reviews*.

[22] Olivieri A, de Angelis S, Dionisi S (2010). Serum transforming growth factor *β*1 during diabetes development in non-obese diabetic mice and humans. *Clinical and Experimental Immunology*.

[23] Zhi W, Sharma A, Purohit S (2011). Discovery and validation of serum protein changes in type 1 diabetes patients using high throughput two dimensional liquid chromatography-mass spectrometry and immunoassays. *Molecular & Cellular Proteomics*.

[24] Han D, Leyva CA, Matheson D (2011). Immune profiling by multiple gene expression analysis in patients at-risk and with type 1 diabetes. *Clinical Immunology*.

[25] Azar ST, Salti I, Zantout MS, Major S (2000). Alterations in plasma transforming growth factor *β* in normoalbuminuric type 1 and type 2 diabetic patients. *Journal of Clinical Endocrinology and Metabolism*.

[26] Abbasi F, Amiri P, Sayahpour FA (2012). TGF-*β* and IL-23 gene expression in unstimulated PBMCs of patients with diabetes. *Endocrine*.

[27] Tesch GH (2007). Role of macrophages in complications of Type 2 diabetes. *Clinical and Experimental Pharmacology and Physiology*.

[28] Jung MR, Min YL, Seung PY, Ho JH (2010). High glucose regulates cyclin D1/E of human mesenchymal stem cells through TGF-*β*1 expression via Ca2^+^/PKC/MAPKs and PI3K/Akt/mTOR signal pathways. *Journal of Cellular Physiology*.

[29] Isono M, Iglesias-de la Cruz MC, Chen S, Hong SW, Ziyadeh FN (2000). Extracellular signal-regulated kinase mediates stimulation of TGF-*β*1 and matrix by high glucose in mesangial cells. *Journal of the American Society of Nephrology*.

[30] Wan YY, Flavell RA (2006). The roles for cytokines in the generation and maintenance of regulatory T cells. *Immunological Reviews*.

[31] You S, Thieblemont N, Alyanakian M-A, Bach J-F, Chatenoud L (2006). Transforming growth factor-*β* and T-cell-mediated immunoregulation in the control of autoimmune diabetes. *Immunological Reviews*.

[32] Franceschi C, Bonafè M, Valensin S (2000). Inflamm-aging. An evolutionary perspective on immunosenescence. *Annals of the New York Academy of Sciences*.

[33] Singh T, Newman AB (2011). Inflammatory markers in population studies of aging. *Ageing Research Reviews*.

[34] McFarlane D, Wolf RF, McDaniel KA, White GL (2011). Age-associated alteration in innate immune response in captive baboons. *Journals of Gerontology A: Biological Sciences and Medical Sciences*.

[35] Willis EL, Wolf RF, White GL, McFarlane D (2014). Age- and gender-associated changes in the concentrations of serum TGF-1*β*, DHEA-S and IGF-1 in healthy captive baboons (Papio hamadryas anubis). *General and Comparative Endocrinology*.

[36] Lin Y, Nakachi K, Ito Y (2009). Variations in serum transforming growth factor-*β*1 levels with gender, age and lifestyle factors of healthy Japanese adults. *Disease Markers*.

[37] Okamoto Y, Gotoh Y, Uemura O, Tanaka S, Ando T, Nishida M (2005). Age-dependent decrease in serum transforming growth factor (TGF)-beta 1 in healthy Japanese individuals; Population study of serum TGF-beta 1 level in Japanese. *Disease Markers*.

[38] Grainger DJ, Mosedale DE, Metcalfe JC (2000). TGF-*β* in blood: a complex problem. *Cytokine and Growth Factor Reviews*.

[39] Saurwein-Teissl M, Blasko I, Zisterer K, Neuman B, Lang B, Grubeck-Loebenstein B (2000). An imbalance between pro- and anti-inflammatory cytokines, a characteristic feature of old age. *Cytokine*.

[40] Bending D, de la Peña H, Veldhoen M (2009). Highly purified Th17 cells from BDC2.5NOD mice convert into Th1-like cells in NOD/SCID recipient mice. *Journal of Clinical Investigation*.

[41] Martin-Orozco N, Chung Y, Chang SH, Wang Y-H, Dong C (2009). Th17 cells promote pancreatic inflammation but only induce diabetes efficiently in lymphopenic hosts after conversion into Th1 cells. *European Journal of Immunology*.

[42] Shao S, He F, Yang Y, Yuan G, Zhang M, Yu X (2012). Th17 cells in type 1 diabetes. *Cellular Immunology*.

[43] Langrish CL, Chen Y, Blumenschein WM (2005). IL-23 drives a pathogenic T cell population that induces autoimmune inflammation. *Journal of Experimental Medicine*.

[44] Mus AMC, Cornelissen F, Asmawidjaja PS (2010). Interleukin-23 promotes Th17 differentiation by inhibiting T-bet and FoxP3 and is required for elevation of Interleukin-22, but not Interleukin-21, in autoimmune experimental arthritis. *Arthritis and Rheumatism*.

[45] Mensah-Brown EPK, Shahin A, Al-Shamsi M, Lukic ML (2006). New members of the interleukin-12 family of cytokines: IL-23 and IL-27 modulate autoimmune diabetes. *Annals of the New York Academy of Sciences*.

[46] Goldberg IJ (2001). Clinical review 124: diabetic dyslipidemia—causes and consequences. *Journal of Clinical Endocrinology and Metabolism*.

